# Arrival timing diagnostics at a soft X-ray free-electron laser beamline of SACLA BL1^1^

**DOI:** 10.1107/S1600577519002315

**Published:** 2019-04-01

**Authors:** Shigeki Owada, Kyo Nakajima, Tadashi Togashi, Tetsuo Katayama, Hirokatsu Yumoto, Haruhiko Ohashi, Makina Yabashi

**Affiliations:** a Japan Synchrotron Radiation Research Institute, Sayo-cho, Sayo-gun 6795198, Japan; b RIKEN SPring-8 Center, Sayo-cho, Sayo-gun 6795148, Japan

**Keywords:** soft X-ray free-electron lasers, arrival timing diagnostics

## Abstract

An arrival timing monitor for the soft X-ray free-electron laser (XFEL) beamline of SACLA BL1 has been developed. A small portion of the soft XFEL pulse is branched using the wavefront-splitting method. The branched FEL pulse is one-dimensionally focused onto a GaAs wafer to induce a transient reflectivity change. The beam branching method enables the simultaneous operation of the arrival timing diagnostics and experiments. The temporal resolution evaluated from the imaging system is ∼22 fs in full width at half-maximum, which is sufficient considering the temporal durations of the soft XFEL and the optical laser pulses.

## Abstract 2

Arrival timing diagnostics between a soft X-ray free-electron laser and synchronized optical laser pulses were performed at SACLA BL1.

## 1. Introduction   

The soft X-ray free-electron laser (XFEL) beamline BL1 of SACLA (Ishikawa *et al.*, 2012 ▸), which employs a dedicated 800 MeV LINAC, started user operation in July 2016. In this beamline, SASE–FEL in the photon energy range 40–150 eV is available with an average pulse energy of ∼80 µJ at 100 eV (Owada *et al.*, 2018*a* ▸,*b* ▸). BL1 has been providing research opportunities for various fields of science, such as materials science (Yamamoto *et al.*, 2018 ▸) and atomic, molecular and optical (AMO) science (Minemoto *et al.*, 2018 ▸).

The recent progress in synchronization techniques has enabled the reduction of the relative arrival timing jitter between FELs and optical lasers to less than 100 fs (Schulz *et al.*, 2015 ▸; Kang *et al.*, 2017 ▸). However, arrival timing diagnostics are still necessary to compensate for the jitter to perform ultrafast pump–probe experiments. In hard XFEL beamlines, transient optical reflectivity or transmissivity changes of semiconductors induced with XFEL irradiation have been probed using a spatial or spectral encoding scheme. A spectral encoding method combining white light with a transmissive target such as a thin film of Si_3_N_4_ has been adopted at hard XFEL endstations of the LCLS (Bionta *et al.*, 2011 ▸; Harmand *et al.*, 2013 ▸). At SACLA BL3, a beam branching method using transmissive grating combined with one-dimensional focusing enabled the performance of pump–probe experiments simultaneously with the arrival timing diagnostics (Sato *et al.*, 2015 ▸; Katayama *et al.*, 2016 ▸). In the extreme ultraviolet (EUV) and soft XFELs, the THz field streaking method with gas targets has been applied (Grguras *et al.*, 2012 ▸; Juranic *et al.*, 2014 ▸; Ivanov *et al.*, 2018 ▸) because it is non-destructive in this photon energy region.

As a beamline instrument, stable and simple operation of the timing monitor is very important. For this purpose, transient transmissivity probed by the spatial encoding manner would be promising. One of the advantages of this scheme is requiring only a fundamental beam of a Ti:sapphire laser without white light or THz generation. While the beam branching method using transmissive optics cannot be simply applied to the EUV and soft X-ray regions, a wavefront splitting scheme is applicable even in those photon energy regions. Recently, we performed a proof-of-principle experiment on the arrival timing diagnostics. Here, a beamline slit and elliptical mirror have been used for extracting a small portion of the incident soft X-ray beam and focusing the beam in one dimension (Owada *et al.*, 2018*a* ▸). Based on the result, we developed an arrival timing monitor for SACLA BL1 that employs the wavefront splitting method. In this paper, we report the design of the timing diagnostics system and the commissioning results including the arrival-timing correlation between the branched beam and the main beam.

## 2. Design   

**Figure 1:**
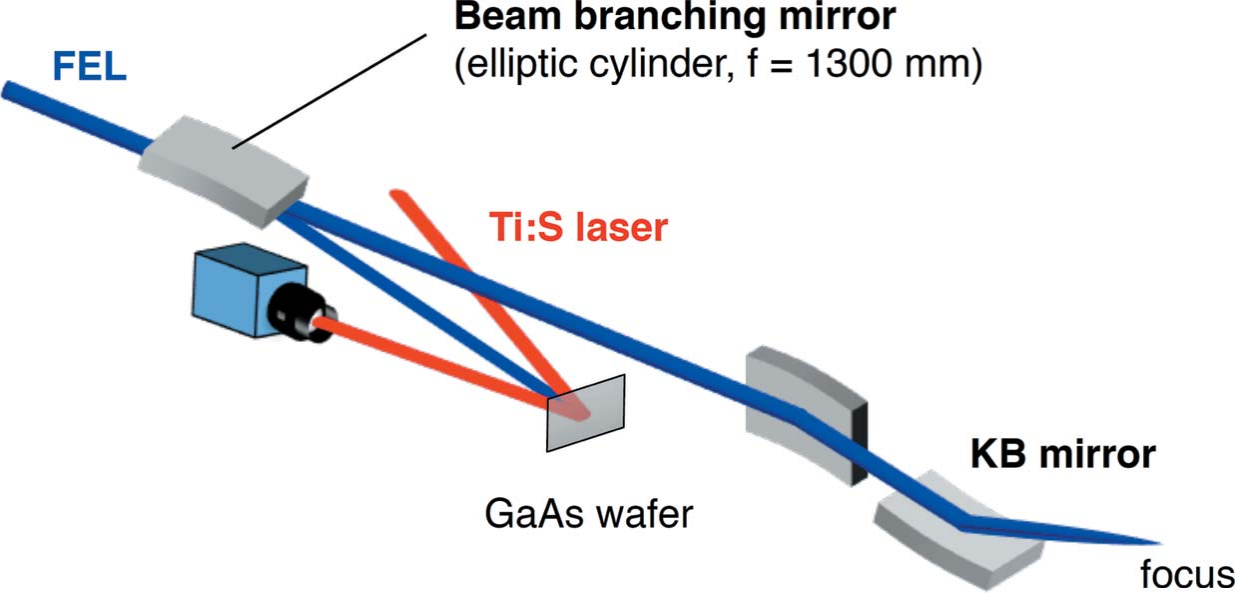
Schematic of the arrival timing monitor at SACLA BL1. The incident soft XFEL beam is introduced to the beam branching mirror. A small portion of the XFEL beam is reflected and focused to a GaAs wafer, while the major part of the beam is introduced to the KB mirror system and focused to the sample position. The optical laser pulses were reflected on the GaAs wafer and imaged onto the visible CCD camera with an imaging lens.

**Figure 2:**
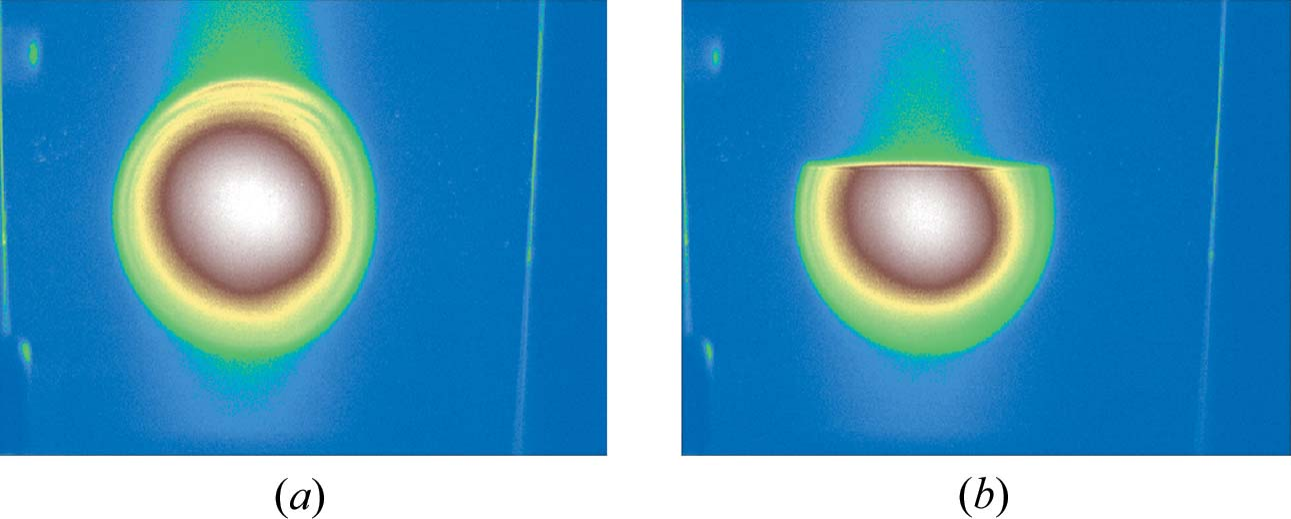
(*a*) Beam profile of the incident soft X-ray pulse at 100 eV. (*b*) Beam profile after inserting the beam branching mirror. The distance between the centre of the incident beam and the mirror edge is ∼5 mm, which corresponds to an ∼15% beam splitting ratio.

**Figure 3:**
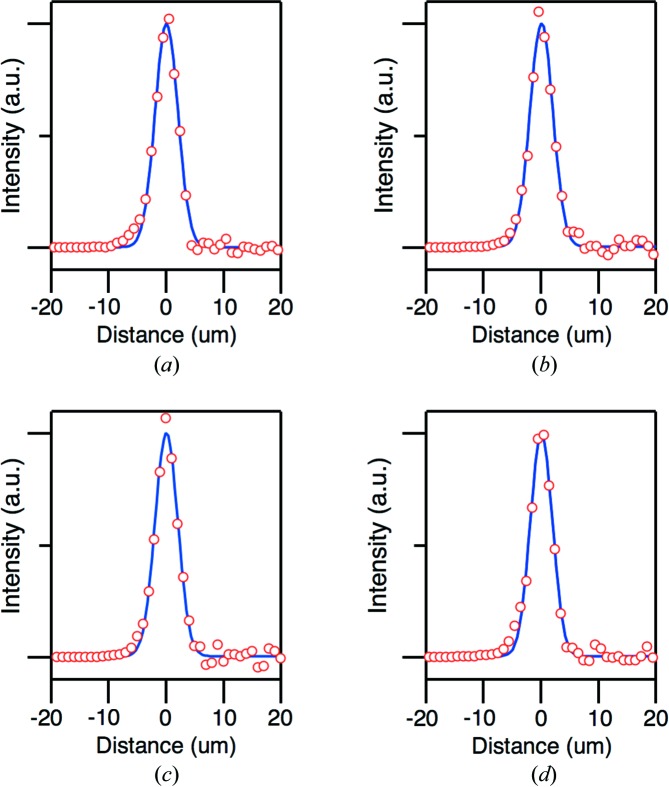
Spatial profiles of the focused beam without beam branching in the (*a*) horizontal and (*b*) vertical directions, measured at *hν* = 100 eV with the knife-edge scan method. The red circles are measured results, while the dashed lines are Gaussian fits. Panels (*c*) and (*d*) represents the focused beam profiles with beam branching in the horizontal and vertical directions, respectively.

A key point of our arrival timing monitor is the combination of the wavefront splitting for beam branching with one-dimensional focusing for efficient pumping of a semiconductor. For this purpose, we designed a new elliptic cylindrical mirror which has a sharp edge of silicon substrate coated with carbon. A schematic view of our arrival timing monitor is shown in Fig. 1 ▸. The upper portion of the incident beam is reflected at a glancing angle of ∼3° and one-dimensionally focused onto the surface of a mirror-polished GaAs wafer, while the remaining lower part is introduced to the endstation EH4a for the experiments. Fig. 2 ▸ shows the soft X-ray beam profile at 100 eV before and after inserting the branching mirror into the incident beam axis. We note that beam branching does not affect the focus size of the KB mirrors installed in the endstation as shown in Fig. 3 ▸.

As a probe pulse, the synchronized Ti:sapphire laser pulses with a 1.57 eV photon energy and a pulse duration of ∼35 fs were one-dimensionally focused using a cylindrical lens. The polarization of the optical laser is horizontal because the reflectivity change ratio of the *p*-polarized optical beam is a few times larger than that of the *s*-polarized beam (Owada *et al.*, 2018*a* ▸). The transient change of reflectivity on the surface of GaAs is detected with a visible CCD camera (OPAL-2000D) combined with a micro-imaging lens (ULWZ-200-IR). The spatial resolution of the camera is 2.1 µm per pixel, which corresponds to a temporal resolution of 5.1 fs per pixel considering the optical geometry of the spatial encoding method.

Since the probe beam irradiated the GaAs wafer at an incident angle of 45°, the focal depth of the imaging lens limits the effective temporal window. Under the current magnification conditions, the effective temporal window is ∼2.5 ps.

## 3. Results   

### 3.1. Single-shot arrival timing diagnostics   

**Figure 4:**
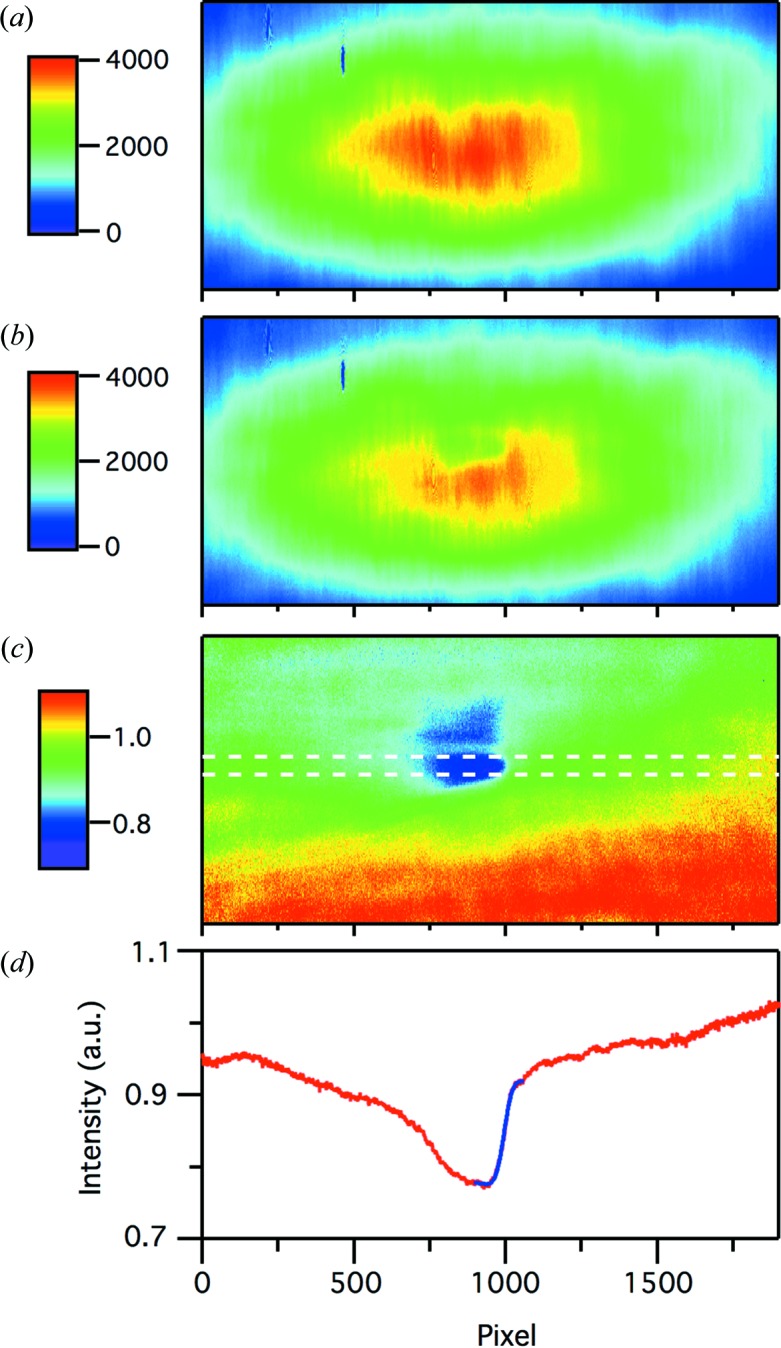
(*a*) The 1000 shots averaged CCD image without XFEL irradiation. (*b*) The single-shot CCD image with XFEL irradiation. (*c*) The single-shot CCD image (*b*) normalized by (*a*). (*d*) Red line: line profile integrated along the vertical axis between white dashed lines in (*c*). Blue line: fitting result using the error function.

At first, we analysed single-shot CCD images to evaluate the arrival timing in the same manner as our previous paper (Owada *et al.*, 2018*a* ▸). At 100 eV with ∼80 µJ of incident pulse energy, ∼15% of the incident energy was branched and focused onto the GaAs wafer. The soft X-ray fluence on the wafer was ∼3 mJ cm^−2^, which was sufficiently high to induce the transient optical reflectivity change and low enough to avoid permanent damage of the GaAs wafer after 12 h irradiation. Under this condition, the edge of reflectivity reduction was detected, as shown in Fig. 4 ▸. While the higher XFEL intensity easily causes permanent damage to the surface of GaAs, the lower XFEL intensity cannot induce sufficient transient reflectivity change, which makes the accurate arrival timing analysis difficult. In order to optimize the XFEL intensity, one can change the branching ratio by varying the vertical position of the branching mirror.

### 3.2. Temporal resolution   

**Figure 5:**
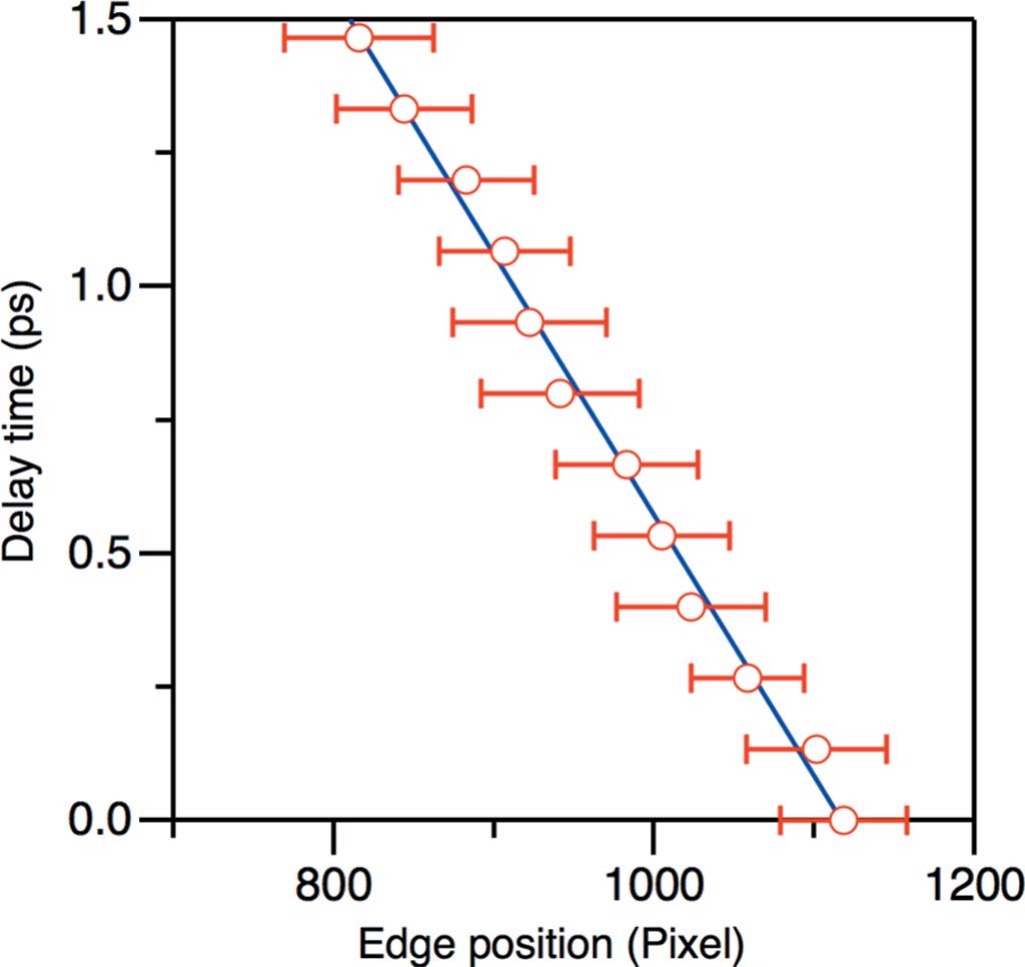
The dependence of 1000-shot-averaged edge position on the CCD images with respect to the delay time between the soft X-ray and the optical pulses. The horizontal bars represent the 2σ width of the edge positions to be evaluated.

The temporal resolution was evaluated by changing the relative delay time between the soft XFEL and the Ti:sapphire laser pulses. Fig. 5 ▸ shows the dependence of the 1000-shot-averaged edge position with respect to the relative delay time. The conversion coefficient from pixel location to relative arrival timing was determined to be 4.9 (2) fs per pixel, which is consistent with that derived from the optical geometry. Although the higher magnification of the imaging lens improves the temporal resolution up to <2 fs per pixel, which was achieved at the timing monitor of SACLA BL3 (Katayama *et al.*, 2016 ▸), the effective time window becomes narrower due to the limitation of the focus depth. The temporal resolution of ∼5 fs per pixel with an effective temporal window width of ∼2.5 ps is an optimized condition considering the relative arrival timing jitter of ∼500 fs (FWHM; Owada *et al.*, 2018*a* ▸), which mainly originated from the synchronization system of the oscillator, and the pulse duration of the soft XFEL and the optical laser pulses, which are estimated to be <100 fs (FWHM) and ∼35 fs (FWHM), respectively.

### 3.3. Correlation measurement   

**Figure 6:**
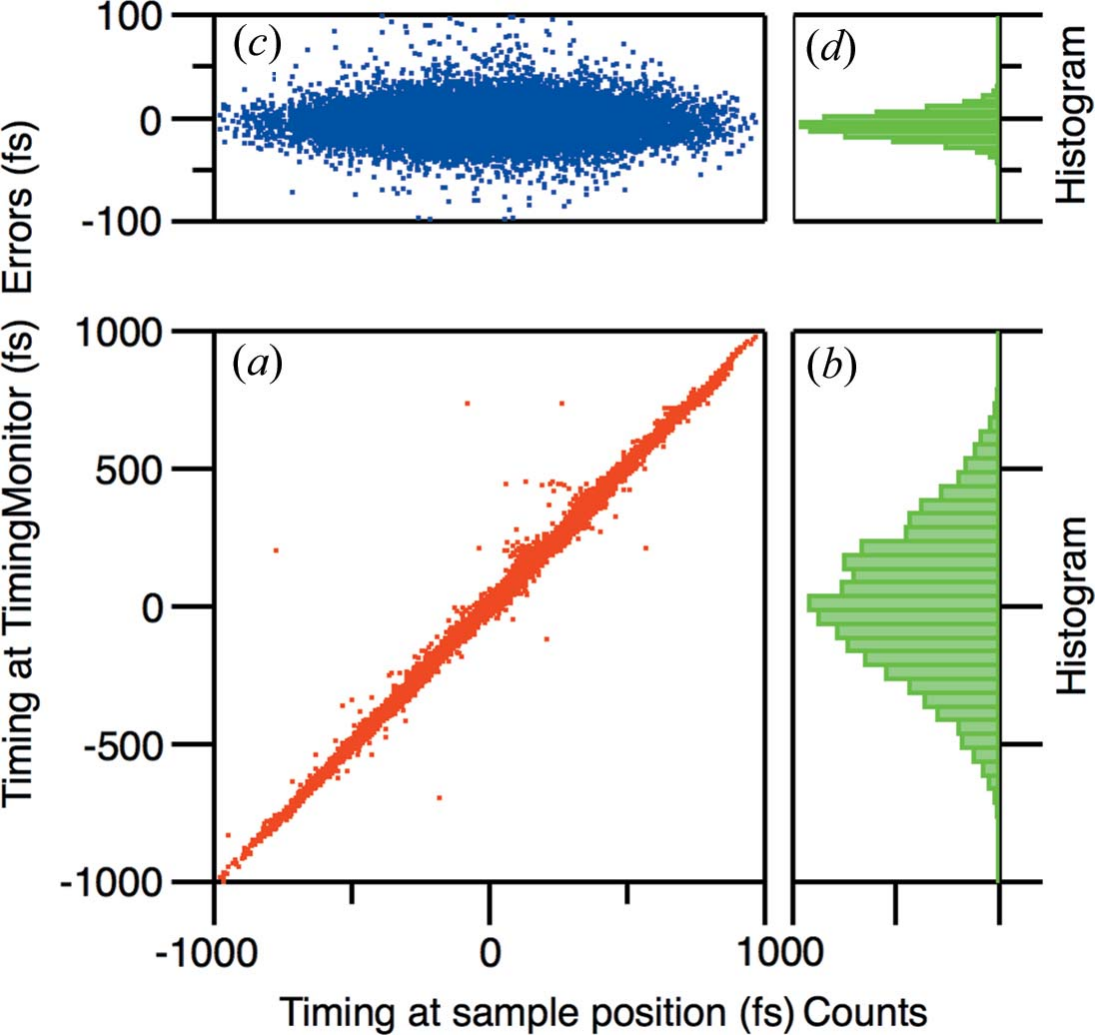
(*a*) Correlation plot between the arrival timings measured at two positions. (*b*) Histogram of the arrival jitter with a bin width of 50 fs. (*c*) Residual errors of the linear fitting of plot (*a*). (*d*) Histogram of plot (*c*) with a bin width of 5 fs.

In order to evaluate the performance of this system, we calculated the correlation by independently measuring the arrival timing at the timing monitor and at the sample position located ∼5 m away from this system. We analyzed 36 000 shots of images and plotted the correlation in Fig. 6 ▸(*a*). The histogram in Fig. 6 ▸(*b*) represents the arrival timing jitter. The residual error of linear fitting, which corresponds to the accuracy of the arrival timing measurement and its histogram, are plotted in Figs. 6 ▸(*c*) and 6 ▸(*d*), respectively. The error width includes the fitting accuracy and systematic errors such as mechanical vibration of the optics and sample. The histogram in Fig. 6 ▸(*d*) shows the FWHM width of 22 fs (*i.e.* RMS width of 13 fs). This means one can achieve ∼20 fs temporal resolution using the arrival timing monitor, which is sufficiently accurate considering the temporal duration of the FEL and the optical pulses.

## 4. Summary   

We have developed the arrival timing monitor for the soft XFEL beamline of SACLA BL1. We adopted the wavefront splitting method for beam branching, which enabled simultaneous operation of the arrival timing diagnostics and ultrafast pump–probe experiments. By measuring the arrival timing correlation between the branched beam and the main beam, an ∼22 fs (FWHM) error is obtained. The arrival timing monitor enables the improvement of the experimental temporal resolution down to a few tens of femtoseconds.
